# Modeling the effect of postgraduate courses on soft skills: a practical approach

**DOI:** 10.3389/fpsyg.2023.1281465

**Published:** 2024-01-05

**Authors:** Luis Alberto Pinos Ullauri, Alexis Lebis, Abir Karami, Mathieu Vermeulen, Anthony Fleury, Wim Van Den Noortgate

**Affiliations:** ^1^IMT Nord Europe, Institut Mines-Télécom, Univ. Lille, Centre for Digital Systems, Lille, France; ^2^Faculty of Psychology and Educational Sciences, KU Leuven, Kortrijk, Belgium; ^3^IMEC Research Group ITEC, KU Leuven, Kortrijk, Belgium; ^4^ICL, Junia, Université Catholique, LITL, Lille, France

**Keywords:** soft skills, courses, modeling, multiple membership, multiple imputation

## Abstract

Over the last decade, Higher Education has focused more of its attention toward soft skills compared to traditional technical skills. Nevertheless, there are not many studies concerning the relation between the courses followed within an academic program and the development of soft skills. This work presents a practical approach to model the effects of courses on soft skills proficiency. Multiple Membership Ordinal Logistic Regression models are trained with real data from students of the 2021, 2022, and 2023 cohorts from the general engineering program in a French Higher Education institution. The results show that attending a postgraduate course in average increases the odds of being more proficient in terms of soft skills. Nonetheless, there is considerable variability in the individual effect of courses, which suggest there can be huge differences between courses. Moreover, the data also suggest great dispersion in the students' initial soft skill proficiency.

## 1 Introduction

Soft skills have gathered considerable attention, especially in Higher Education Institutions (HEI). International organizations, such as the European Union with the Bologna process, have pushed HEI to consider competencies, life-learning skills or soft skills in their curricula (European Higher Education Area, 2016; Council of the European Union, 2018). The work by Caeiro Rodriguez et al. (2021) studied the European perspective of soft skill development in Higher Education investigating the different practices in 5 European countries, describing the current pedagogical methodologies (e.g., problem, project, or simulation-based methods) that aim to support soft skills, their advantages and limitations. Moreover, Almeida and Morais (2021) analyzed 4 case studies of HEI in Portugal addressing soft skills in their curricula. They realized that although the number of courses that explicitly took into account soft skills in their pedagogical activities was rather small, there was growing pressure and interest from HEI in incorporating such skills as core parts of the curricula.

Arvanitis et al. (2022) studied, through focus groups with stakeholders from HEI in Greece, strategies to fill the soft skills “gap.” This is the difference between the soft skill proficiency demanded in industry compared to the one developed in HEI. A possible strategy for HEI towards reforming and changing curricula across all levels and degrees could be to analyze their current state, that is, how their curricula help students develop their soft skills. Since courses are amongst the most common modular parts of any curriculum (there could be other elements that award credits such as projects or internships), they could serve as valuable information to help explain the students' soft skill proficiency across time. This would allow the quantification of the effect of the courses of the curricula on the students' soft skills. In other words, we could inspect whether a particular course helps more or less, compared to other courses, the students in terms of their soft skills. Therefore, in this context, the following research questions are examined.

RQ1: How can we model the effect of courses on the soft skills of postgraduate students?
Secondary research questions are:
RQ2: How is the soft skill proficiency's behavior throughout the academic program?
RQ3: How is the behavior of the course effects on soft skills?
RQ4: What are the degrees of linear correlation of course effects across different soft skills?

Nevertheless, before attempting to answer any of these questions, it is important to underline that there is not a full consensus regarding the definition of “soft skills.” The work by Touloumakos (2020) describes how these expanded from the overall definition of “skills”, encompassing a variety of attributes and traits, which do not require specific contexts in contrast to more technical skills. Their increasing relevance has promoted many researchers to try to define them, engaging in a long debate about what may or may not be considered as soft skills (Hurrell et al., 2013). According to Almonte (2021), soft skills are difficult to conceptualize in a cohesive and clear way without considering their elasticity and multifaceted perspectives. Furthermore, there are other terms that may be pseudo-interchangeable (depending on the context and definition) with soft skills such as 21st century skills (Partnership for 21st Century Skills, 2007), 4C Skills (Ye and Xu, 2023), graduate skills (Barrie, 2007), competencies, life skills (Council of the European Union, 2018), generic skills (Tuononen et al., 2022), transferable skills, and employability skills.

These other terms, however, may focus on other aspects, which do not necessarily concern soft skills. For example, employability skills generally relate to skills that help increase the chances of employment whereas transferable skills stress the skills that can be developed in a particular situation and transferred into another (Jardim et al., 2022; van Ravenswaaij et al., 2022). On the other hand, graduate skills emphasize the skills expected from university graduates whereas generic skills mostly relate to the generic aspect of skills that are not context-specific and can be used in different situations. For the purposes of this study, the term “soft skills” refers to non-technical, personal and social skills that can be used in various situations. For instance, Leadership and Stress Management are non-technical skills with a personal and interpersonal nature. In a situation, such as an industrial interdisciplinary project, the participants may need to take full advantage of these skills to manage their stress and lead the groups they are in charge of. Moreover, these same skills could also be used on academic settings, such as group coursework within courses where students would need to work together to accomplish the requirements of the pedagogical activity.

The pressure of the focus on soft skills has not only been present in HEI, but also in industry. There are several studies investigating the requirements of the industry in terms of soft skills along with the perceptions of both student and employers of soft skills as means to improve graduate employability (Ellis et al., 2014; Succi and Canovi, 2020; Xu et al., 2022). Moreover, the work by Cacciolatti et al. (2017) studied the clash between the employers' needs of both technical and soft skills along with the universities' policies towards these skills in the United Kingdom.

Since skills, in general, are latent variables (i.e., variables that can not be directly observed, and only estimated with a model that links latent variables with one or more manifest variables), the estimation of soft skills is an important part to consider. There are some strategies that may be followed to estimate soft skills. A questionnaire consisting of binary or multiple choice questions, designed to estimate a soft skill in particular (e.g., Stress Management), could be deployed. Then, either Classical Test Theory (CTT) (Novick et al., 1968), or Item Response Theory (IRT) (Lord, 1952; Rasch, 1960) could be used. With CTT, the questionnaire could be validated [e.g., with item-total correlations and Cronbach's alpha (Novick and Lewis, 1966)] in order to ensure that the questions reliably measure the construct, and then estimate the soft skill with the assumption that the computed score comprises the true soft skill proficiency alongside an error residual. In contrast, IRT models in a non-linear way the probability of correct responses based on underlying person and item characteristics, being the person characteristic, in this case, the soft skill proficiency. Another strategy could be to develop a marking scheme or rubric that describes in depth the levels of proficiency that are expected of the individuals to be assessed, thereby having pre-defined ordered levels of proficiency.

Several studies have estimated soft skills or their perception through some of the previously mentioned strategies. For instance, Chamorro-Premuzic et al. (2010) performed various studies in United Kingdom universities, where one of those was based on the students' perceptions of the influence of soft skills proficiency towards obtaining first class degrees (a distinction degree awarded at United Kingdom universities). They used a 7-likert type scale (scale from 1 to 7, where 1 was “Not at all” and 7 “Extremely useful”) of an inventory of 15 soft skills, and computed a total score per soft skill by calculating the mean score across items. Other studies have also taken a similar approach towards the estimation of soft skills with the computation of likert-type scale-measures across various items (Feraco and Meneghetti, 2022; Salem, 2022). Moreover, the work by Zendler (2019) studied the modeling of competencies in Computer Science Education using IRT. Furthermore, multidimensional IRT (Adams et al., 1997; Reckase, 2009), which considers the interplay between multiple abilities through compensatory or partially compensatory methods, has been used to model competencies (Hartig and Höhler, 2008, 2009), and could be used, in principle, to estimate latent soft skills based on soft skill item responses.

There have also been a few studies which investigated the interplay between courses and soft skills. The work by Muukkonen et al. (2022) studied the students' self-perceptions towards competence gains in 28 courses from two large Finnish universities. The study showed the effects of courses on skill growth to be statistically significant between courses. We follow this line of thought, in which students, besides achieving technical learning objectives, may also develop their soft skills by attending courses. We further assume that this development occurs in varying degrees across the entire curriculum (given their multidisciplinary nature, multiple courses could affect the same soft skill, e.g., a course from computer science, such as mobile application development, and another course from chemical engineering, such as organic chemistry, could both affect the problem solving skills of students). However, optimizing the development of soft skills throughout the program requires understanding the relation between courses and soft skills. An exploration of the effect of courses on soft skills can serve as a tool to study whether it is appropriate to adjust the curriculum (e.g., add, remove or adapt courses with soft skills centered pedagogical activities) if the curriculum does not sufficiently foster soft skills according to the aims of the HEI.

Continuous data is comprised within an infinite number of possible measurements between two specified limits. These measurements can include decimals. For instance, a continuous scale where the minimum is 1 and maximum is 4 can comprise measurements such as 1.01 or 3.85. Continuous models are based on distributions that can predict continuous data. Nonetheless, these models can be adapted to account for the discrete nature of categorical data. Soft skill proficiency can be estimated categorically (e.g., Leadership could be assessed in levels of proficiency such as Satisfactory, Good, Very Good, and Excellent). A first modeling approach could be to use mixed regression models to explain the soft skill proficiency with courses as predictors and random effects across students regarding their individual soft skill traits. In this approach, there would be a fixed effect, same for all students, of each course towards the students' soft skills proficiency. Nonetheless, a sizeable dataset would be needed to obtain stable fixed course effect estimates, particularly in HEI where the number of courses offered to students is considerably high. Therefore, in order to account for this issue, an overall general fixed effect of courses can be estimated instead of individual course effects. Moreover, random effects of courses and students can also be included to consider their variability. Students may not begin the program with the same level of soft skill proficiency. Similarly, courses may differ between themselves towards their effects on soft skills. For instance, course *x* may be more effective in helping students develop Project Management skills compared to course *y*.

The general aim of this study is to explore the relation between courses and soft skill proficiency of students, and showcasing a practical approach to model these course effects, which could serve as valuable input for the overall analysis of soft skills development within an academic program. In order to so, we leverage longitudinal data from a French engineering school, IMT Nord Europe, which is interested in redesigning their current general engineering program by incorporating soft skills. The engineering school developed a marking scheme to estimate the soft skills proficiency of the general engineer profile. This marking scheme is used throughout the academic program after each of the students' yearly internships, providing a longitudinal dataset describing their progress across time in terms of soft skill proficiency.

Section 2 describes in more detail the data used in this study as well as the statistical treatment of missing data from a part of the 2021 cohort. The models are also presented along with our Bayesian approach to estimate the parameters.

Section 3 presents the descriptive results of the students' soft skills proficiency across the last three years of the engineering program. Afterwards, the estimates of the parameters are presented, both continuously in the logit scale and through odds.

In Section 4, a discussion regarding the results is presented. Finally, the conclusions, limitations and possible alternatives for future work are described in Section 5.

## 2 Materials and methods

### 2.1 Data

The data was collected from the cohorts of students that graduated in 2021, 2022, and 2023 of the general engineering program (884 students in total), at IMT Nord Europe. The 5 year general engineering program contemplates 5 internships (one per year) for the students. Besides their regular studies, the students are also in constant involvement with the industry throughout the entire length of the program. After each internship, the students have their soft skills proficiency assessed by internship tutors with the previously mentioned marking scheme (see Table 1), which considers 10 soft skills and classifies their proficiency in 4 ordered categories. The marking scheme was developed by IMT Nord Europe in 2016, and later deployed by their Professional Development department in 2019.

**Table 1:** Translated soft skill assessment scheme.

**Skill**	**Description**	**Score**
Problem solving	Solve problems in familiar environments or in known contexts. Use solutions that are already outlined	1
	Solve related problems in new environments or in unknown contexts. Find solutions adapted to each situation	2
	Solve problems that are not always well defined, in complex environments or in contexts subject to strong constraints	3
	Reformulate problems according to different constraints and find adapted and efficient solutions	4
Innovation and creativity	Contribute to the search for ideas and solutions by participating in exchanges and creativity sessions and propose improvements	1
	Propose and apply proven solutions to new or different contexts	2
	Design and implement new solutions with a view to efficiency	3
	Recognize, transmit and implement the conditions and processes for generating innovation	4
Organization	Work independently on the basis of indications and instructions given. Monitor performance indicators, detect and report problems within the scope of activity	1
	Prioritize and plan their own workload, evaluate and correct completed activities (use performance indicators to assist in decision making)	2
	Prioritize and establish actions according to the stakes of the activities. Set up new relevant indicators. Share and promote best practices	3
	Transmit and share methods of organization and rigor with his interlocutors. Encourage them to use and follow relevant performance indicators. Deploy continuous improvement plans	4
Agility and adaptability	Implement activity changes that are requested	1
	Adapt and re-prioritize their activities and organization to changes and constraints	2
	Evaluate the impact of changes and propose appropriate responses or solutions	3
	Anticipate future developments and changes	4
Interaction and communication	Listen actively, express and formalize a point of view clearly, share information. Reformulate an idea without distorting it	1
	Effectively present an argument in a logical and argued manner, both in writing and orally. Know how to use a vocabulary that goes beyond the usual	2
	Use expression techniques (written and oral) adapted to the message to be delivered and the target audience (specialists and/or non-specialists), in a clear and unambiguous manner	3
	Communicate skilfully (vocabulary, style, ...) and finely in complex situations (sensitive message, difficult audience, unexpected situation...)	4
Project management	Work within a group (a team) and collaborate with team members in an open manner by communicating feedback on work	1
	Manage a small independent project or a part within a larger program. Accompany one or two collaborators on an operational activity	2
	Lead a major project or coordinate several operational projects simultaneously. Lead a complete team on an operational activity or project	3
	Lead and coordinate several strategic or operational projects simultaneously, training teams on project management, setting up adapted animation devices. Settling conflicts and arbitration situations in an objective and factual way without breaking interpersonal relations	4
Conviction	Explain a point of view in a clear way and with predefined or prepared arguments. Listen to, understand and reproduce a need expressed by others	1
	Adapt one's behavior, attitude, speech and arguments according to the audience in order to maximize the quality of exchanges. Interact to reformulate and deepen one's need in order to specify it and propose a response	2
	Identify and decipher the positions of the different strategic audiences, anticipate their expectations and reactions, identify and reach the right influence relays with the people to be convinced. To be a force of proposal in relation to an expressed need while rallying the stakeholders	3
	Implement strategic actions in a complex environment in a relevant and recurring manner to convince and influence key players. Anticipate the needs of their “clients,” their environments and guide them in their evolution	4
Stress management	Work in low stress situations	1
	Adapt themselves to temporary stressful situations	2
	Adapt to prolonged stress	3
	Use strategies to deal with stress themselves and for their collaborators	4
Leadership and influence	To take a step back and take initiatives in the service of activities and collaborators belonging to a close circle	1
	Share their own vision with familiar and occasional (or close and temporary) collaborators	2
	Promote their vision to internal and external decision-makers, and encourage their teams to take the initiative	3
	Pool resources and partners by creating a dynamic around a strategy and/or a change process	4
Decision making	Makes decisions based only on rules	1
	Makes decisions in situations where rule interpretation is possible	2
	Makes decisions interpreting the rules and improves them	3
	Makes complex decisions in absence of rules	4

The assessment scheme was developed by Cecile Leroy, Jean-Luc Caenen, and Didier Urschitz.

Moreover, the students share the same curriculum up until the end of the third year. In the fourth and fifth years, they must choose a specialization, from which there is a catalogue of courses (104 different courses in the dataset, where some are exclusive to specific specializations) to choose with no prerequisites other than the hard skills we expect they have developed in previous courses. In addition, there are courses which are transversal and can be chosen across all specializations. The specializations are Energy and Environment, Digital, Industry and Services, and Materials and Civil Engineering. By taking advantage of the soft skill assessments at the end of the third year as an initial reference, we can focus our interest in the last two years (year 4 and 5), where the students can freely choose their courses (a maximum of 11 courses are followed up until the end), and therefore may end up with different courses by the end of the program. If the students were to follow the exact same curriculum, the variability in soft skill proficiency could be explained by a number of factors such as the initial differences between students or how a same course affects differently students. Nonetheless, in this case where students may follow different courses, their variability in terms of soft skill proficiency could also be affected by their heterogeneous course history.

We categorized the years as stages (*Stage* = 0 at the end of third year and reference for the models, *Stage* = 1 at the end of fourth year and *Stage* = 2 at the end of the fifth year or academic program). Table 2 shows the number of students, whose soft skill proficiency data was assessed, per student cohort. It can be seen that there is no data from the 2021 cohort at the initial stage (the soft skill assessment program had not yet started) along with the last ongoing stage of the 2023 cohort. Given the importance of much data in order to model the effects of courses on soft skills, statistical techniques are used to handle the missing data from stage 0 of the 2021 cohort. As the same data will be collected in future years, we plan to update the analyzes each year.

**Table 2:** Number of students per stage and cohort.

**Stage**	**Cohort 2021**	**Cohort 2022**	**Cohort 2023**
0	-	194	339
1	250	245	366
2	141	141	-

The dataset structure, which comprises the longitudinal annual soft skills proficiency and course history of the last three years of the general engineering program, is shown in Table 3. The first column represents the student identifiers. The *Stage* describes the year during which the data from the student in question were collected whereas the *W*_*c*_ columns represent dummy variables with *w*_*cst*_ equal to 1 if student *s* has followed course *c* by stage *t*, otherwise equal to 0. Moreover, the dummy variables keep the previous courses up until stage *t*. The *N* column describes the cumulative number of courses of student *s* up until that time *t* (It could also be thought of as the row sum of the *W*_*c*_ columns). *sskill*_*ist*_ is the soft skill score of student *s* at stage *t* on soft skill *i*. For instance, the student with identifier 884 by stage 2, followed a total of *N*_*s* = 884, *t* = 2_ = 11 courses, amongst which were courses 1, 2, and 104, and was assessed on soft skill 1 and 10, respectively, with scores of 4 and 3.

**Table 3:** Dataset structure.

**Student id**	**Stage**	***W*_1_**	***W*_2_**	**..**.	***W*_103_**	***W*_104_**	***N***	***sskill*_1_**	**..**.	***sskill*_10_**
1	0	0	0	...	0	0	0	2	...	1
1	1	0	1	...	1	0	6	3	...	2
1	2	1	1	...	1	0	11	3	...	3
2	0	0	0	...	0	0	0	1	...	2
2	1	1	0	...	0	0	4	3	...	3
...	...	...	...	...	...	...	...	...	...	...
883	1	0	1	...	0	0	5	3	...	2
883	2	0	1	...	1	0	11	3	...	4
884	0	0	0	...	0	0	0	1	...	2
884	1	0	1	...	0	0	6	3	...	2
884	2	1	1	...	0	1	11	4	...	3

It is important to note that aside from the missing data at stage 0 from Cohort 2021, there were cases of soft skill scores which were not assessed during the internships, and were therefore treated as missing data for the models.

### 2.2 Modeling approach

Since our outcome of interest is an ordinal variable (i.e., ordered categorical data such as the levels of soft skill proficiency from Table 1), we use ordinal logistic regression. Its general form is presented Equation 1, where *Y* is a variable with *K* categories. The cumulative probability *P*(*Y* ≤ *k*) is the probability of *Y* of being in category 1, 2,..., or *k*. Furthermore, the odds of *Y* being less or equal to a *k* category is expressed by the ratio $\frac{P\left(Y\leq k\right)}{P\left(Y>k\right)}$. This model allows us to predict the probability, via a logit link function, of the *K* categories, totalling 100% with all the categories, based on the linear combination of the explanatory variables *W*.


(1) $$\begin{array}{c} logit\left(P\left(Y\leq k\right)\right)=\ln\;\left(\frac{P\left(Y\leq k\right)}{P\left(Y>k\right)}\right)=\alpha_{k0}-\overset{n}{\underset{i=1}{\sum }}\beta_{i}w_{i} \end{array}$$

where *k* = 1, 2, ..., *K* − 1

The α_*k*0_ intercepts represent the *K* − 1 thresholds needed to compare against the linear combination of effects [the probability of the last category *P*(*Y* = *K*) is calculated by the complement, 1 − *P*(*Y* ≤ *K* − 1)]. The β parameters are fixed effects that, in our case, could very well describe the individual effects of courses, with the *W* being dummy binary variables that determine whether the students followed or not that particular course by that time. Nonetheless, this model would use only fixed effects, same for all students, and because some students may begin with different soft skill proficiency levels, random intercepts can be considered to account for the student's individual characteristics [i.e., by adding a random residual θ_*s*_ to the intercepts α_*k*0_, with θ_*s*_~*N*(0, σ_θ_*s*__)]. In this way, the probability of a student being assessed a soft skill proficiency category *k* would be a function of both the student's individual characteristic and the course history. Nonetheless, due to the relatively small size of the dataset compared to the amount of courses that can be chosen by students, it may be quite difficult to adequately estimate the effects of individual courses (β_1_, …, β_*n*_). Therefore, we consider a general course effect, and a random deviation of individual courses from this mean course effect. These random deviations are assumed to follow a random normal distribution. Because each of the courses can contribute to the soft skill proficiency, a multiple membership model was used (Hill and Goldstein, 1998).

Multiple membership models arose from the need of properly modeling data structures which do not accommodate traditional hierarchical data clusters. For instance, in our case, the soft skill scores for Leadership of student *s* belong to that student and no one else. This means that those scores are nested within students. Nonetheless, the same cannot be said regarding the relation between students and courses because students would follow multiple courses (and not just one), where each of them would have an effect on the students' soft skill scores. The students would therefore belong to multiple courses. Therefore, Multiple Membership Ordered Logistic regression models are used to explain the cumulative probability of soft skill proficiency of soft skill *i* from student *s* and stage *t* being classified in category *k*. It is important to remark that the linear combination of effects is performed in the logit scale, and can be either interpreted with odds or continuously in the logit scale. In addition, it is assumed the course effects remain the same throughout time (regardless of lecturer or syllabus changes) in order to limit the complexity of the models. Moreover, the models are uni-dimensional, in the sense that each soft skill is modeled separately thereby having an ensemble of 10 models explaining each a soft skill in particular.

Equation 2 describes the model where α_0*ik*_ represents the various thresholds against which the linear combination of effects are compared. β_*i*_ is the fixed average course effect (same for all students) on soft skill *i*, and *N*_*st*_ the number of courses followed by student *s* and stage *t*. *u*_*ic*_ is the random effect of course *c* on soft skill *i* whereas *w*_*cst*_ represents the contribution weights of the course random effects on soft skill *i*. Finally, θ_*is*_ is a random effect across students towards soft skill *i*.


(2) $$\begin{array}{c} logit\left(P\left({sskill}_{ist}\leq k\right)\right)=\alpha_{0ik}-\beta_{i}N_{st}-\overset{C}{\underset{c=1}{\sum }}u_{ic}w_{cst}-\theta_{is} \end{array}$$

where *k* = 1, 2, 3 represents the soft skill proficiency categories. Regarding the weights, *w*_*cst*_ = 1 if student *s* followed course *c* by stage *t*, otherwise it is equal to 0. Moreover, the random effects are assumed to follow normal distributions, *u*_*ic*_~*N*(0, σ_*ic*_) and θ_*s*_~*N*(0, σ_*is*_).

**Figure 1:**
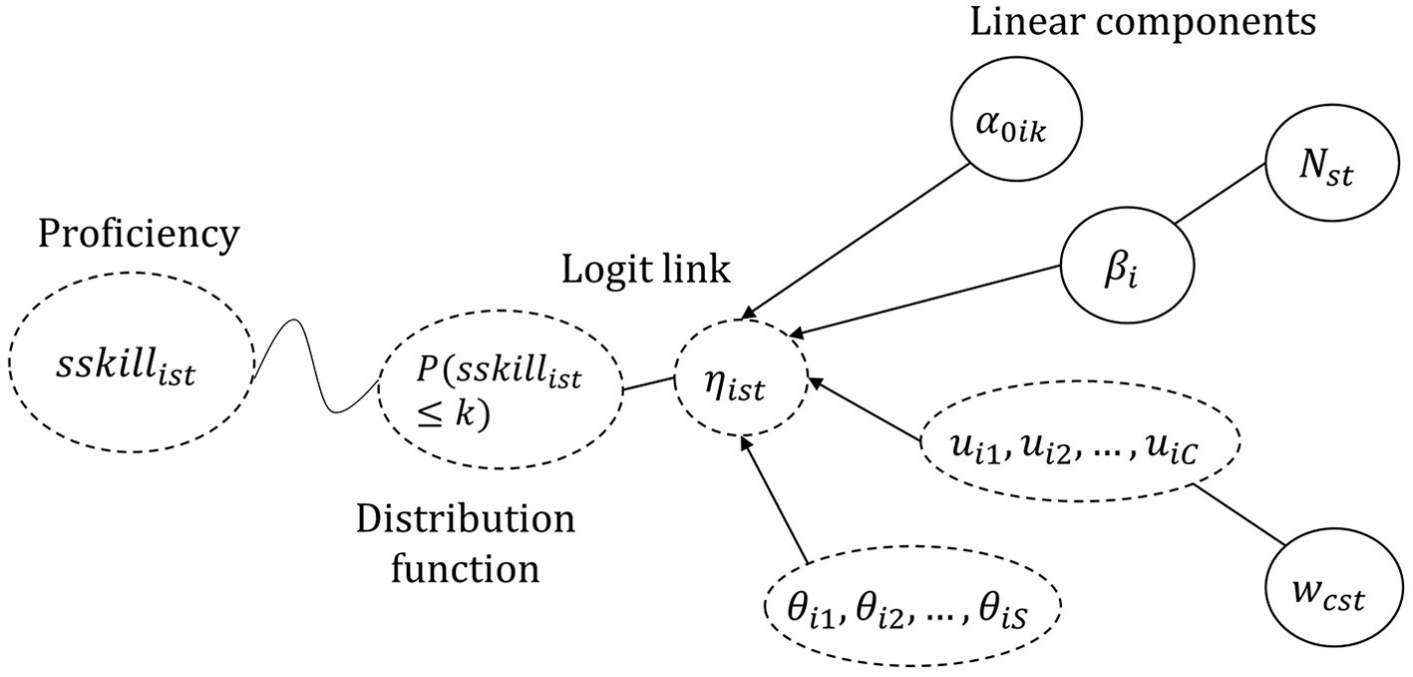
Schema of the model. The dotted circles represent random variables whereas the non-dotted circles represent fixed parameters and variables. η_*ist*_ is the linear combination of the α thresholds with the general fixed effect of the average course along with the random effects of courses (*u*_*ic*_) and students (θ_*is*_).

Figure 1 shows a graphical representation of the model, following the visualizations of Generalized Linear Mixed Models by De Boeck and Wilson (2004). The probability of the soft skill of a student being assessed a certain proficiency category depends on the the linear combination, η_*ist*_, linked to the expected outcome by a logit link function. This η_*ist*_ results from the interplay between fixed and random variables from the courses as well as the individual characteristics of student *s*.

According to the model in Equation 2, if a student followed 4 courses with identifiers (7, 12, 29, 44), the general fixed effect β_*i*_ towards soft skill *i* would be multiplied by 4 and 4 random effects (one for each course identifier) would contribute to the overall effect of courses for this student. Since the weights of the random effects would be equal to 1 for those courses, the overall effect could be expressed as β_*i*_ + *u*_*i*7_ + ⋯ + β_*i*_ + *u*_*i*44_. This means course effect *c*_*i*_ could be explained by the average course effect plus their individual random effects β_*i*_ + *u*_*ic*_*w*_*cst*_.

A previous study by Arreola and Wilson (2020) utilized a similar approach to the one of our models, albeit their models predicted dichotomous categorical academic performance, and not soft skills proficiency. They defined the outcome as success if students achieved a Grade Point Average greater than 2 or 3. Moreover, their models considered the students as members of multiple instructors or lecturers (where each student may have been affected by one or multiple instructors along their studies), whereas in our particular case, the students are considered multiple members of courses. This means that students follow various courses, and the students' course history varies in both in number and type (identifier) of courses.

### 2.3 Bayesian analysis

A Bayesian approach was used considering its flexibility in terms of model implementation. The software *Stan* by Carpenter et al. (2017), the R programming language (R Core Team, 2021), and the R package *RStan* by Stan Development Team (2023) were used to code, compile and fit the models to the data.

Since we have no previous data regarding course effects on soft skills, non-informative (i.e., priors that do not affect the posterior given their non-informative nature) and weakly-informative (i.e., priors that have a mild influence on the posterior distribution in order to keep the estimates in sensible ranges) were considered for all parameters. Table 4 shows the parameters priors, which were chosen based on the default priors in the R package *brms* by Bürkner (2018). A standard deviation σ = 2.5 is used for the priors of the standard deviations σ_*is*_ and σ_*ic*_ for both course and student random intercepts. It is important to add that σ_*is*_ and σ_*ic*_ are restrained to be positive in the Bayesian implementation.

**Table 4:** Non-informative and weakly-informative priors for the model parameters.

**Parameter**	**Prior distribution**
β_*i*_	*U*(−∞, +∞)
σ_*is*_	*t*(*df* = 3, μ = 0, σ = 2.5)
σ_*ic*_	*t*(*df* = 3, μ = 0, σ = 2.5)
*u*_*ic*_	$N\left(\mu =0,\sigma =\sigma_{ic}\right)$
θ_*is*_	$N\left(\mu =0,\sigma =\sigma_{is}\right)$

Furthermore, the models were fitted with 4 chains, where each chain ran for 5,000 iterations and 1,000 burn-out samples, having a total of 16,000 post warm-up iterations per model fit. The models were fitted for each of the ten soft skill dimensions, resulting in several model fits. The empirical estimate $\hat{R}$ was equal to 1 for all fits, which suggests convergence.

### 2.4 Multiple imputation

In our experiment, multiple imputation was used to impute 5 times the soft skills missing data from stage 0 of the 2021 student cohort. The missing data from that stage and cohort comprised the soft skill scores. The imputation was made possible with a model that depends on the data from further stages (as well as stage 0 from the 2022 and 2023 cohorts) comprising the student and soft skill scores. Multiple imputation is a highly popular approach to handle missing data (Rubin, 1987, 1996). It comprises a range of techniques that allow to generate values in order to fill in the missing data, based on other known variables from the dataset. We used the R package *mice* (van Buuren and Groothuis-Oudshoorn, 2011), which allows flexible implementations of various multiple imputation methods, such as predictive mean matching [imputation method proposed by Rubin and Schenker (1986); Little (1988) and used in our study]. By imputing several times the missing data, we end up with several datasets. These datasets would be similar with the only difference being the generated values corresponding to the 2021 students at stage 0. It is also important to note that by having multiple datasets, there would be various estimations (1 for each dataset) of the courses effects on the same soft skill dimension *i*. In this case, a course *c* would have 5 effects for soft skill *i*. Rubin's rule (Rubin, 1987), which was proposed to pool parameter results from multiple imputation, is used further in Section 3 to combine the different parameter estimates from the imputed datasets, and to account in the standard errors of the regression parameters for the uncertainty about the imputed values.

While the computational cost of multiple imputation is relatively low, the cost of estimating the model parameters with each of the imputed datasets is considerable. In a computer Intel(R) Core(TM) i7-10810U CPU @ 1.10GHz 1.61 GHz and 32 GB of RAM, the Bayesian estimation needed approximately 35 to 40 min to fit a model (by running the chains in parallel) given the current number of parameters and size of the dataset. This means that in order to estimate the effects (40 min × 5 imputed datasets × 10 soft skills), 2,000 min or approximately 33 h were necessary. Therefore, the number of imputed datasets was chosen to keep the overall computational cost feasible. Nonetheless, a higher number of imputations, in principle, would provide more robust estimations.

## 3 Results

Before interpreting the results, it is important to note that it should be done with caution given the complexity of the data as well as the considerable number of parameters to estimate, associated with a relatively low number of students (in comparison).

### 3.1 Descriptive results

#### 3.1.1 Soft skill proficiency

**Figure 2:**
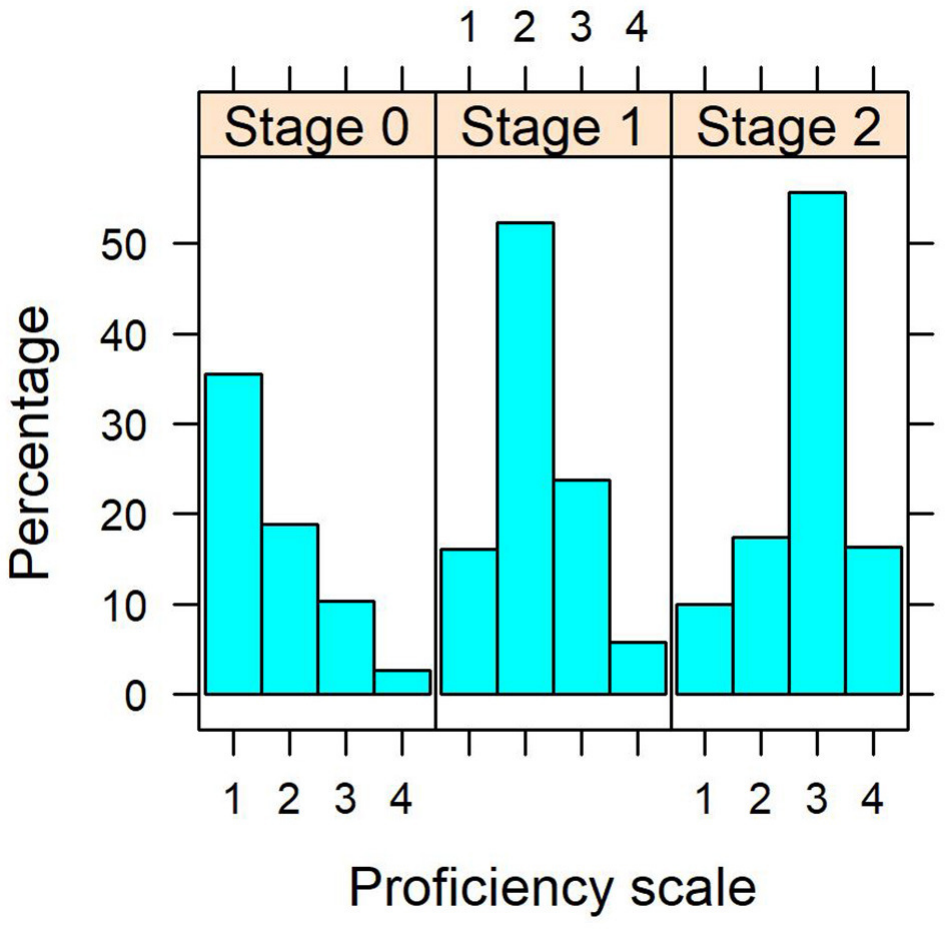
Organization skills proficiency histograms across stages. Please note proportions may not amount to 100% due to missing data from the assessments aside from the imputed data at stage 0 from Cohort 2021.

The discrete distribution of all soft skill dimensions have similar tendencies regarding the probability of higher proficiency towards the end of the program. An example is shown in Figure 2, which depicts the histograms of the students' organization skill proficiency across stages. It can be seen that the distribution at stage 0 tends to be right skewed (more probability of the first soft skill scores), whereas stage 1 is less skewed and appears to be somewhat symmetric (more probability in the 2nd and 3rd levels). On the other hand, stage 2 tends to have a left skewed distribution (higher probability of the last categories and lower on the first levels). This may suggest the probability of higher scores increases across stages.

A more detailed descriptive analysis of the soft skill proficiency across time is shown in Table 5. It can be seen that the highest average proficiency was observed for problem solving with average values of 2.43, 2.92, and 3.10 (on the 4-point scale) across the three stages. The lowest average proficiency was observed, at stage 0, for Organization, followed closely by Leadership. It can also be seen that several soft skills have lower standard deviations across the stages (e.g., Decision Making with 0.87 at stage 0, 0.74 at stage 1 and 0.66 at stage 2), although the decreasing trend presents itself in varying degrees. Moreover, the average change (Δ_*t*+1,*t*_) depicts the difference between the average proficiency at stage *t* + 1 and *t*. It can be noted that all these changes are positive, albeit some are very small at certain stages.

**Table 5:** Average, standard deviation, and average changes Δ_*t*+1*t*_ of the soft skill scores across stages.

**Soft skill**	**Stage 0** **Avg (SD)**	**Δ_*t*1*t*0_**	**Stage 1** **Avg (SD)**	**Δ_*t*2*t*1_**	**Stage 2** **Avg (SD)**
Problem solving	2.43 (0.80)	+0.49	2.92 (0.77)	+0.18	3.10 (0.75)
Innovation	1.86 (0.97)	+0.57	2.43 (0.78)	+0.44	2.86 (0.74)
Organization	1.70 (0.87)	+0.50	2.20 (0.78)	+0.59	2.79 (0.84)
Adaptibility	1.96 (0.89)	+0.43	2.39 (0.77)	+0.59	2.99 (0.72)
Communication	2.30 (0.83)	+0.58	2.87 (0.81)	+0.10	2.96 (0.79)
Project management	1.83 (0.63)	+0.16	2.00 (0.67)	+0.35	2.35 (0.83)
Conviction	2.08 (0.65)	+0.12	2.20 (0.73)	+0.45	2.65 (0.76)
Stress management	2.11 (0.83)	+0.73	2.84 (0.70)	+0.10	2.94 (0.70)
Leadership	1.73 (0.74)	+0.53	2.26 (0.71)	+0.34	2.60 (0.73)
Decision making	1.97 (0.87)	+0.41	2.38 (0.74)	+0.53	2.91 (0.66)

### 3.2 Multiple membership ordinal logistic regression results

Table 6 presents the pooled mean results from the imputed datasets on each of the soft skills. It can be seen that the average effect of attending an additional course in logit scales ranges from 0.11 in Communication to 0.27 in Leadership. This also means that for each average course (course whose effect does not deviate from the mean effect β_*i*_, *u*_*ic*_ = 0) that an average student (student whose random intercept is equal to the 0 mean amongst all students, θ_*is*_ = 0) follows, maintaining the other variables constant, the odds of having gained proficiency in leadership after attending the course (*e*^0.27^ = 1.30 or 30% increase) are greater than communication (*e*^0.11^ = 1.12 or 12% increase).

**Table 6:** Pooled mean parameter results from the imputed dataset model fits (with the standard errors SE in parantheses).

**Soft skill**	**β (SE)**	**Odds**	**σ_*c*_**	**σ_*s*_**
Problem solving	0.18 (6.24e-5)	1.19	0.15	0.34
Innovation	0.24 (1.08e-4)	1.27	0.13	0.43
Organization	0.23 (4.38e-5)	1.26	0.16	0.36
Adaptability	0.22 (1.34e-4)	1.25	0.13	0.42
Communication	0.11 (7.42e-5)	1.12	0.24	0.36
Project management	0.14 (9.21e-6)	1.14	0.14	0.26
Conviction	0.14 (7.94e-5)	1.15	0.12	0.31
Stress management	0.25 (2.11e-5)	1.29	0.26	0.24
Leadership	0.27 (0)	1.30	0.10	0.32
Decision making	0.23 (2.86e-5)	1.26	0.11	0.38

Please note that all effects are in log-odd scales except the odds.

**Figure 3:**
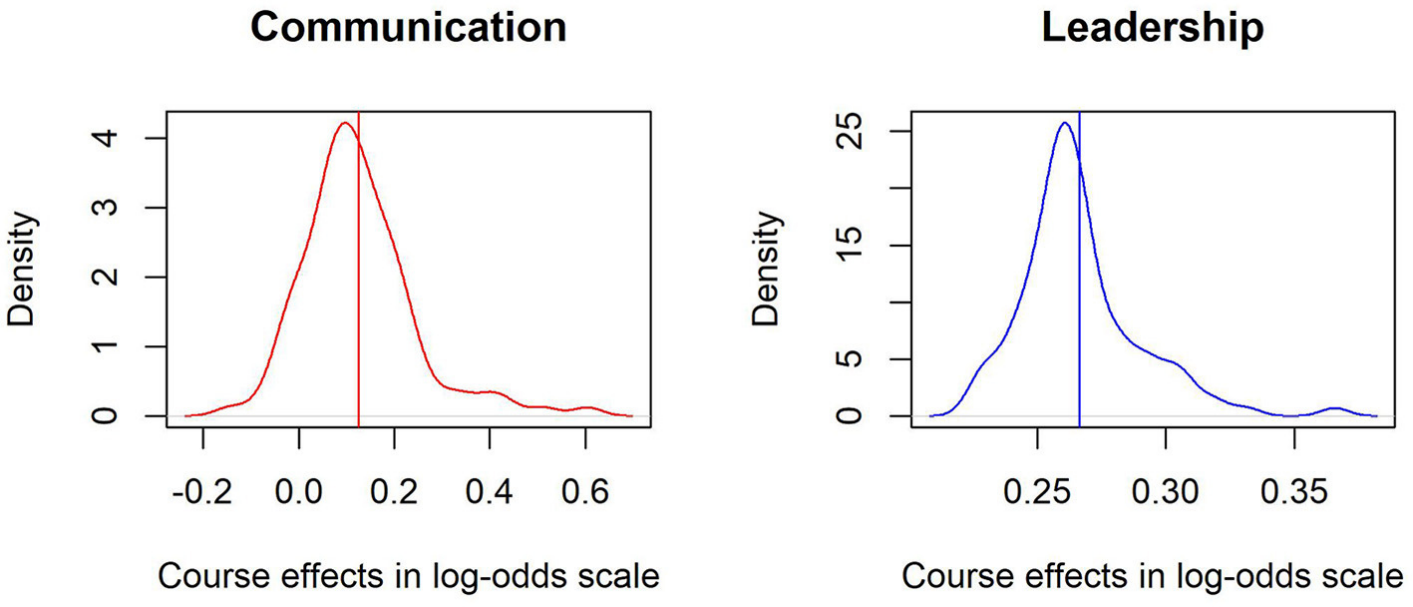
Kernel density plot of the course effects over Communication and Leadership skills. The vertical lines describe the pooled mean average course effect in the logit scale whereas the dispersion represents the variability amongst the individual effects of courses.

The standard deviations of the random effects of the courses range from 0.10 in Leadership to 0.26 in Stress Management. These values are considerably high given that β_*i*_ is measured on a logit scale. Given the assumption of a normal distribution approximately 95% of the course effects would be comprised within 1.96 standard deviations of the mean. For the case of Leadership, whose mean effect is 0.27, this interval would be defined between 0.074 and 0.466 [0.27 − 1.96(0.10) = 0.074, 0.27 + 1.96(0.10) = 0.466]. Moreover, there are some soft skills such as Communication where the interval would comprise a substantial part bellow zero, which further suggests there can be huge differences between courses. Although on average there is a positive effect of attending a course, not all courses have such positive effects. Figure 3 shows the kernel density distributions of the random effects across courses towards Communication (red) and Leadership skills (blue) centered around their average course effects (β). First, it can be seen that both distributions resemble normal distributions, albeit Communication distribution reaches negative values in logit scales, which further suggest not all courses seem to have positive effects. Furthermore, the highest standard deviation of the random effects correspond to the students, where the minimum is 0.24 in Stress Management and the highest 0.43 in Innovation, suggesting even greater variability amongst students in terms of their initial soft skill proficiency.

**Figure 4:**
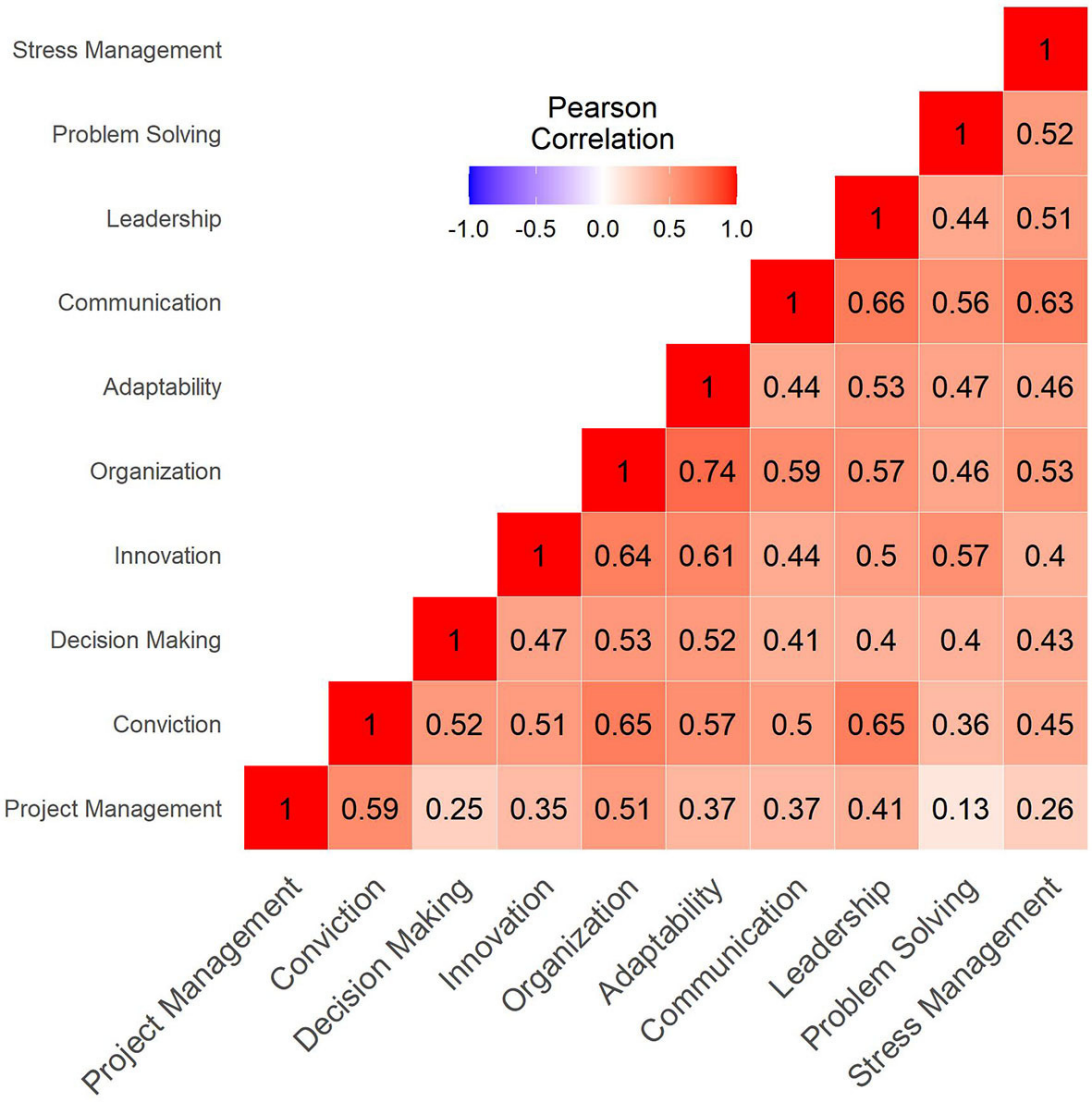
Correlation matrix of course random effects across soft skill dimensions. The higher the correlation the more red it becomes. Similarly, the less correlated, the less red it turns. Perfect linear correlations are depicted as fully red.

Additionally, Figure 4 shows a heat map of the Pearson correlation between the course random residual effects across the various soft skill dimensions. It can be seen that all soft skill dimensions are positively correlated, which means the course effects help develop in general all dimensions. Nonetheless, the coefficients display varying degrees of correlation. While several of the dimensions are considerably correlated (ρ_*sskill*_*x*_*sskill*_*y*__ > 0.40), there are a few dimensions with lower correlation such as Project Management and Problem Solving(ρ_*sskill*_*x*_*sskill*_*y*__= 0.13), Project Management and Decision Making (ρ_*sskill*_*x*_*sskill*_*y*__= 0.25) and Project Management and Stress Management (ρ_*sskill*_*x*_*sskill*_*y*__= 0.26). There are also some highly correlated dimensions such as Organization and Adaptability (ρ_*sskill*_*x*_*sskill*_*y*__= 0.74), Communication and Leadership (ρ_*sskill*_*x*_*sskill*_*y*__= 0.66), and Organization and Conviction (ρ_*sskill*_*x*_*sskill*_*y*__= 0.65).

## 4 Discussion

The descriptive results suggest a positive evolution of the average soft skill proficiency of the engineering students across their curriculum. The discrete probability distribution of the soft skill proficiency changes throughout time, being positively skewed at the beginning of the course free-choice part of the program. This means that at the end of the third year, most students are in the first levels of soft skill proficiency. However, by the end of the program, the distribution becomes negatively skewed, and most students tend to be in the last levels of proficiency, which is mostly a good sign for the academic program.

The model results show Leadership to be the most positively affected soft skill by an average course, followed closely by Innovation and Stress Management. Moreover, the least positively affected soft skill is Communication, followed by Project Management. Furthermore, there is a considerable dispersion of course effects, which may suggest not all courses help students develop their soft skills with the same intensity. The correlation matrix of the random effects show that the course effects on soft skills are positively related, though there are varying degrees of correlation. This may suggest not all courses focus their pedagogical activities evenly on all soft skills. For instance, the modest correlation coefficient between Project Management and Problem Solving (ρ_*sskill*_*x*_*sskill*_*y*__= 0.13) suggest courses that strongly promote one of those soft skills may not necessarily support the other soft skill with the same intensity.

While other approaches such as Charoensap-Kelly et al. (2016) and Muukkonen et al. (2022) focus, respectively, on the perception of competence gain of individual courses and the change of behavior after following a training program, our approach is centered around the relation between courses and the soft skill proficiency of the students. That is, how the course history of the students can help us explain their soft skill proficiency. In addition, our approach leveraged the longitudinal assessments of soft skill proficiency of students throughout the academic program. Nevertheless, it is important to note that the assembly of the dataset, which comprises the longitudinal history of both soft skills and courses from 884 engineering students from 3 different cohorts, involves considerably high costs regarding logistics, time (at least the last 3 years in this case of a cohort of students), management, communication, data cleaning and pre-processing. Additionally, the importance of the dataset became even stronger given the non-availability of public datasets of a similar nature.

Nevertheless, there are several limitations to consider. First, we have to be prudent to interpret the estimated course effects as causal effects, since students were not randomly assigned to courses. The students chose their own courses, making the comparability amongst student groups a bit difficult. If the proficiency of a student group that followed a course increased considerably, we cannot say that this is due to the course itself. It can be due to other kinds of activities that the students performed on top of the course (and that they would have done, even if they had followed another course). Second, the soft skill assessment is performed only once per year, making the dataset size quite difficult to enlarge longitudinally (in terms of soft skill assessments across time) unless these were performed between semesters. Third, due to convergence issues it is currently impossible to include fixed effects of courses as well as additional random effects corresponding to specializations and tutors (4 effects of specialization, their standard deviation, and 1608 tutors alongside their variance, which provides a total of 1,612 additional random effects and 2 variance parameters). Third, in favor of simplicity it is assumed the course effects remain the same regardless of lecturer, syllabus or pedagogical design changes across time, which may not necessarily be the case for most institutions. Fourth, HEI in Europe may contemplate semesters abroad (e.g., Erasmus exchange programs), which makes the integration of course history data across multiple institutions quite difficult.

## 5 Conclusions and future work

In this work, we answered our research questions by proposing the use of Multiple Membership ordinal logistic regression models to allow us to understand the relation between attending postgraduate courses and their effects towards the soft skill proficiency of students. Moreover, we have shown practical methods that may provide great insight for HEI (such as IMT Nord Europe) interested in adapting their curriculum towards soft skills development. It is our hope that this article inspires practitioners and other researchers to further explore and propose other methodologies to model the effect the postgraduate courses on soft skills.

There are several alternatives that could be considered for future work. First, the addition of random intercept effects per specialization, which is not currently included due to the size of the dataset. Second, the inclusion of random intercept effects across internship tutors, who assess the students' soft skills proficiency. Third, the addition of the internship organization, and possibly categorize it by type (e.g., technological, pharmaceutical, clothing) in order to analyze whether the type of organization affects differently the soft skill proficiency of the students. Fourth, the study of the self-perception of their soft skills, and analyze whether these self-assessments correlate to the tutor-assessments. Finally, another strategy could be to study the relation between soft skills and not the courses themselves, but the pedagogical designs they are based on (e.g., problem, project, or simulation-based), which could reduce the complexity, and also provide valuable feedback for curriculum analysis. Nonetheless, it would require more work in discerning which methodologies are applied, and how close they are between the different courses in order to adequately define the weights of the multiple membership design, where students would be multiple members of one or various methodologies.

## Data availability statement

The original contributions presented in the study are included in the article/supplementary material, further inquiries can be directed to the corresponding author.

## Ethics statement

Ethical approval was not required for the study involving humans in accordance with the local legislation and institutional requirements. Written informed consent to participate in this study was not required from the participants or the participants' legal guardians/next of kin in accordance with the national legislation and the institutional requirements.

## Author contributions

LP: Conceptualization, Data curation, Formal analysis, Methodology, Writing – original draft, Writing – review & editing, Validation. AL: Methodology, Writing – review & editing. AK: Methodology, Writing – review & editing. MV: Methodology, Writing – review & editing. AF: Funding acquisition, Methodology, Supervision, Writing – review & editing. WV: Formal analysis, Methodology, Supervision, Validation, Writing – review & editing.
